# Supplementary material for: Adaptations in Plasmodium tubulin determine distinct microtubule architectures, mechanics and drug susceptibility
Source: Nat Commun. 2026 Mar 5;17:2275. doi: 10.1038/s41467-026-70181-0 (PMC12966321; doi:10.1038/s41467-026-70181-0)
Supplement: Supplementary file 1 — Supplementary Information [file 41467_2026_70181_MOESM1_ESM.pdf]

Supplementary Information

Adaptations in *Plasmodium* tubulin determine distinct microtubule architectures, mechanics and drug susceptibility

Mamata Bangera [1,‡], Jiangbo Wu [2], Daniel Beckett [2], Dominik Fachet [3,4], Josie L. Ferreira [5], Gregory A. Voth [2], Simone Reber [3,6*], Carolyn A. Moores [1*]

[1] Institute of Structural and Molecular Biology, Birkbeck, University of London, UK
[2] Department of Chemistry, Chicago Center for Theoretical Chemistry, Institute for Biophysical Dynamics, and James Franck Institute, The University of Chicago, Chicago, IL, USA
[3] Max Planck Institute for Infection Biology, Berlin, Germany
[4] IRI Life Sciences, Humboldt-Universität zu Berlin, Berlin, Germany
[5] Institute of Structural and Molecular Biology, University College, London, UK
[6] Berliner Hochschule für Technik, Berlin, Germany

‡ Current address: Indian Institute of Technology Madras, Chennai, India
* Joint corresponding authors
Simone Reber: ORCID ID: 0000-0002-5287-2332
E-mail: reber@mpiib-berlin.mpg.de
Carolyn A. Moores; ORCID ID: 0000-0001-5686-6290
E-mail: c.moores@bbk.ac.uk

Supplementary Figures 1-7

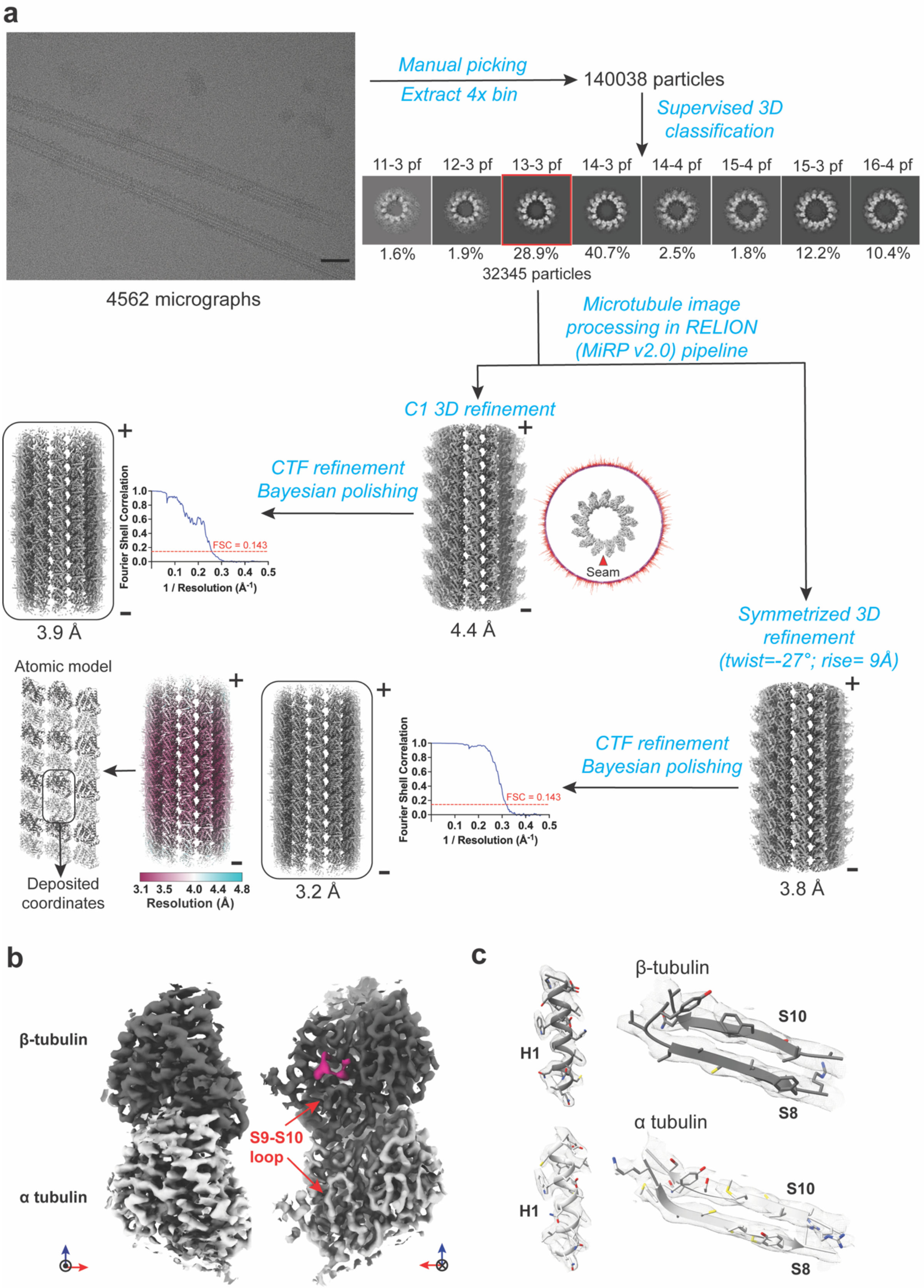

Supplementary Fig. 1: Cryo-EM reconstruction of *P. falciparum* kinesin-8B motor domain-bound paclitaxel-stabilized microtubules. a, Cryo-EM data processing workflow.

Steps of the pipeline are indicated in blue. A representative micrograph is shown. Scale bar = 50 nm. Cross-sections of classes obtained from reference-based 3D classification have been shown with corresponding architectures specified above. EM reconstructions are shown in grey with plus and minus ends of the microtubules labeled. Round-edge boxes highlight the 3D electron density maps that have been deposited to the EMBD. Angular distribution of particles used in C1 3D refinement are represented as cylindrical histogram plots around the microtubule (top view) and the seam is indicated with a red arrowhead. Corresponding FSC plots and maps coloured by local resolution are also shown. The fitted atomic model is shown in cartoon representation, and the coordinates for a tubulin dimer outlined by a round-edge box are deposited to the PDB. **b,** Cryo-EM density of a *P. falciparum* tubulin dimer segmented from the 13-protofilament paclitaxel-stabilized microtubule reconstruction, viewed from outside (left) and lumen (right). Density for the α-tubulin, β-tubulin and paclitaxel is shown in light, dark grey and magenta respectively. **c,** Representative regions of the density (mesh) are shown with the fitted model (cartoon representation with side chains).

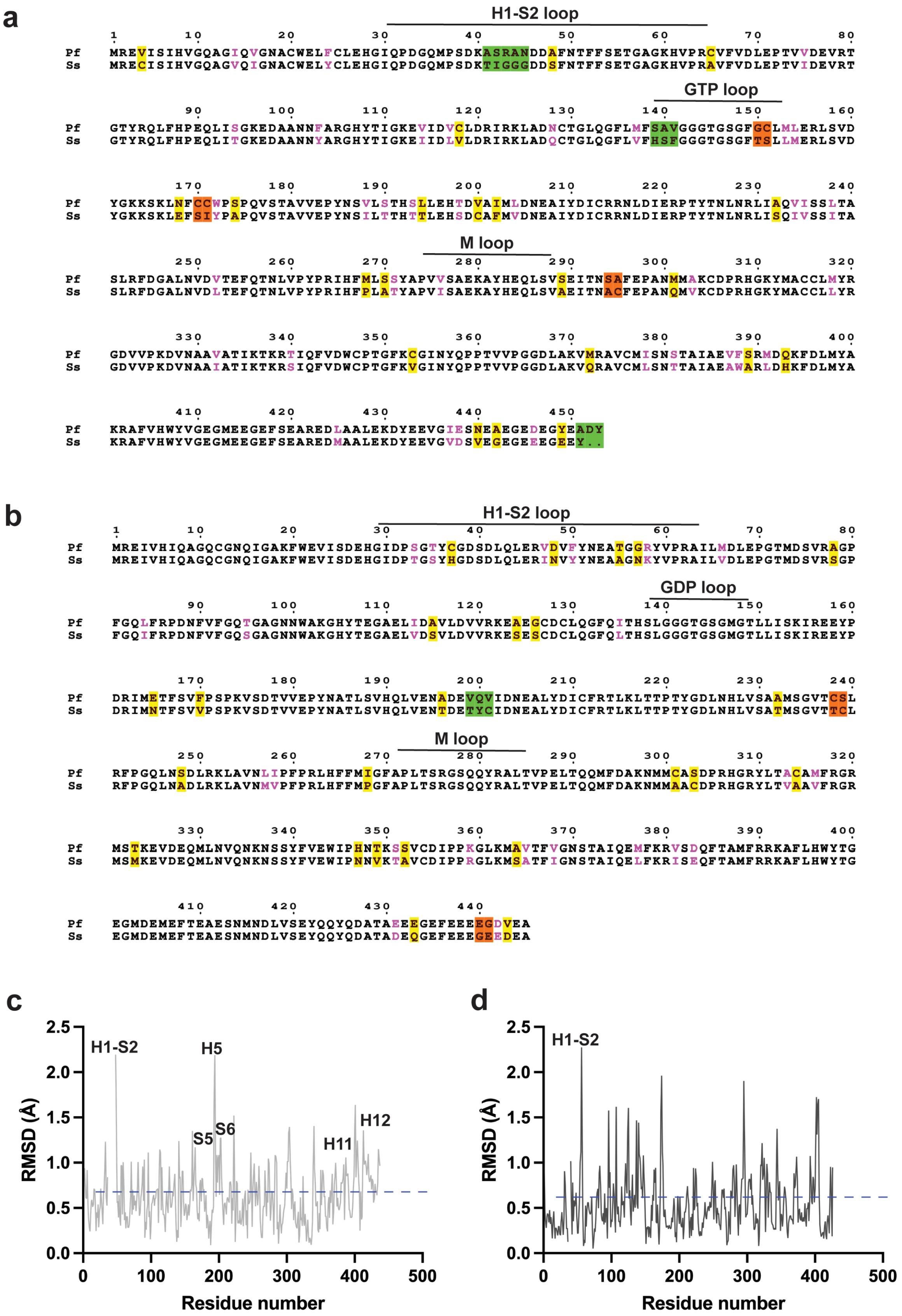

Supplementary Fig. 2: Sequence and structural comparison between *P. falciparum* and brain tubulin. a and b, Sequence alignment of *P. falciparum* and brain α- and β-tubulin

(Uniprot IDs- *Pf* α1-tubulin: Q6ZLZ9; *Pf* β-tubulin: Q7KQL5; porcine α1B-tubulin: Q2XVP4; porcine β-tubulin: P02554) respectively. Similar residues have been coloured in pink. Non-identical residues have been highlighted based on the number of consecutive residues (yellow-1 residue; orange- 2 residues; green-3 or more residues). Key sites in the tubulin dimer have been marked over the sequence. **c and d,** C-α backbone RMSD plots obtained from structural superposition of *P. falciparum* and brain α- and β- tubulin respectively. The blue dotted line indicates the average RMSD.

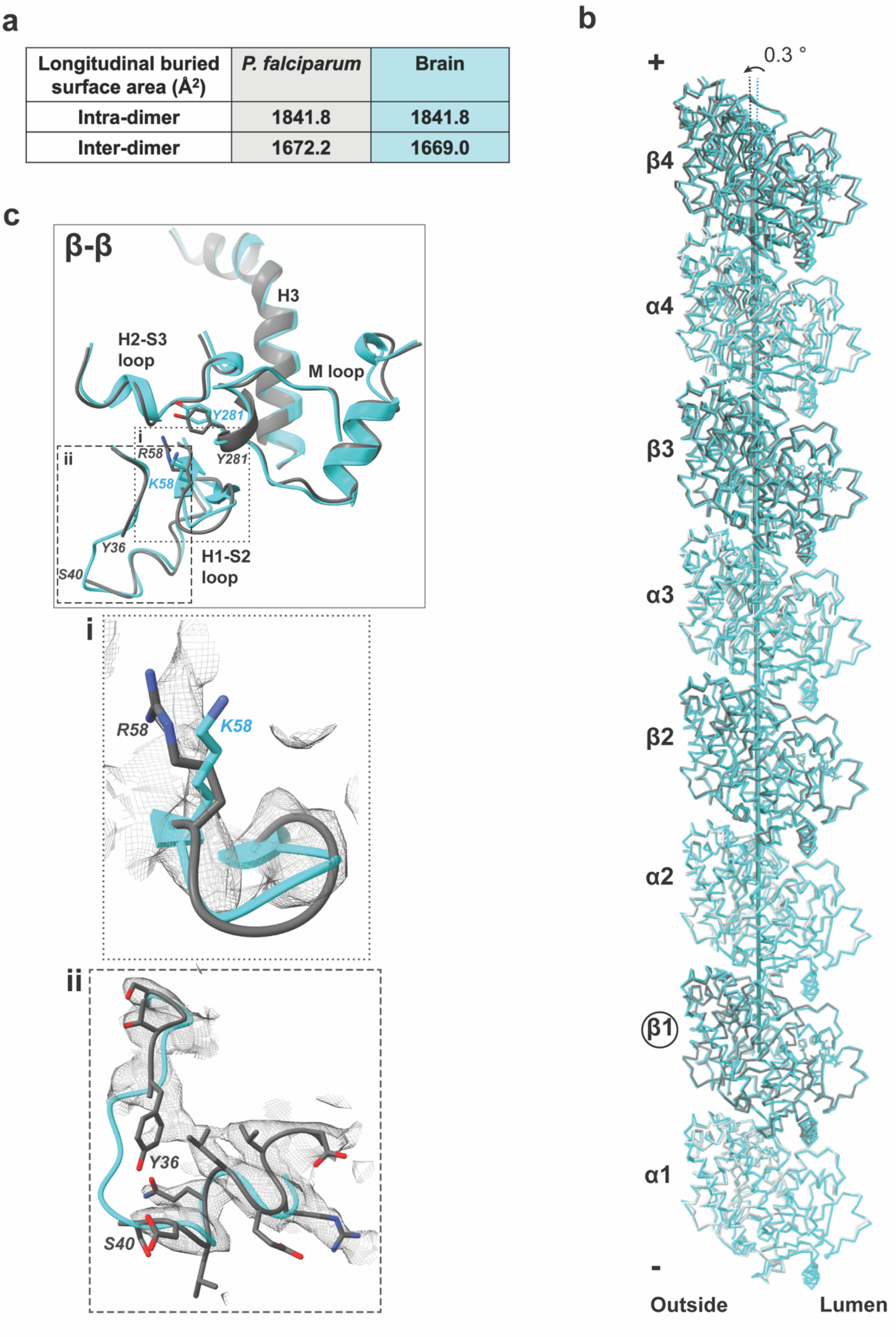

Supplementary Fig. 3: Differences in longitudinal and lateral interfaces of P. *falciparum* and brain microtubules. a, Comparison of buried surface areas of longitudinal interfaces in *P. falciparum* and brain microtubules. **b,** Twist in protofilament from *P. falciparum*

microtubule as compared to brain model (porcine α1B- and β-tubulin; PDB ID: 5SYF) visible in 4th tubulin dimer from the bottom (*P. falciparum* α-tubulin: light grey; *P. falciparum* β-tubulin: dark grey; brain α- and β- tubulin: cyan). **c,** Comparison of β-β lateral interactions between *P. falciparum* and brain microtubules. The interacting loops are depicted as cartoon Cα backbone models (*P. falciparum* β-tubulin: dark grey and brain β-tubulin: cyan) and side chains for residues Arg58 and Tyr281 in *P. falciparum* and Lys58 and Tyr281 in brain β-tubulins are indicated. **Insets** Segmented cryo-EM density for selected regions at the *P. falciparum* β-β interface outlined by dotted (i) and dashed (ii) lines, shown as a grey mesh.

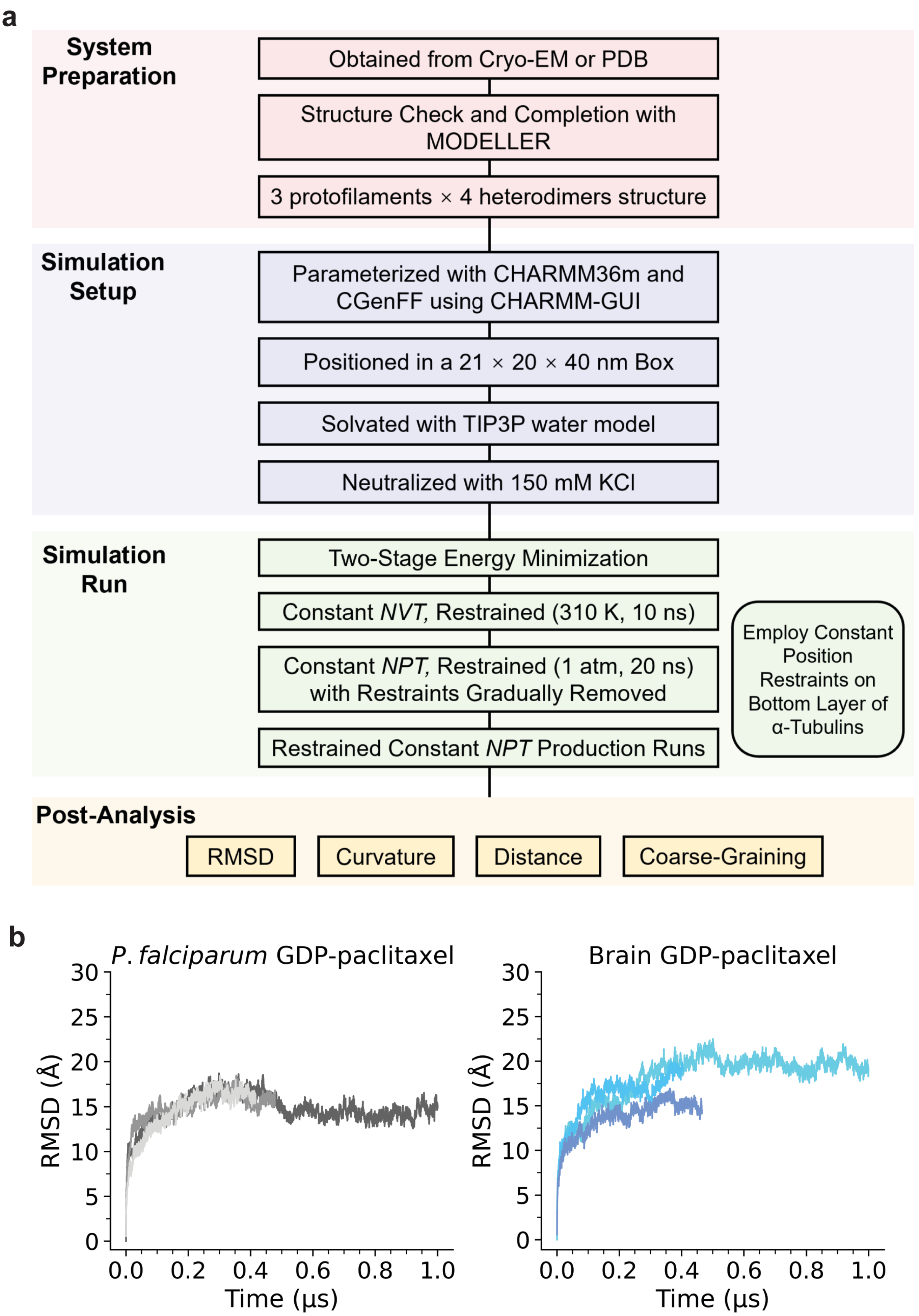

Supplementary Fig. 4: Dynamics of *P. falciparum* and brain microtubule ends. a, Workflow for MD simulations **b,** Root mean square deviation (RMSD) profiles of Cα atoms

for three replicate runs of *P. falciparum* (grey) and brain (blue) microtubule (porcine α1B- and β-tubulin; PDB ID: 5SYF) lattice patches in GDP-paclitaxel-bound states over 1 μs molecular dynamics simulation time. The RMSD stabilizes after approximately 300-400 ns for both systems, indicating equilibration of the structures.

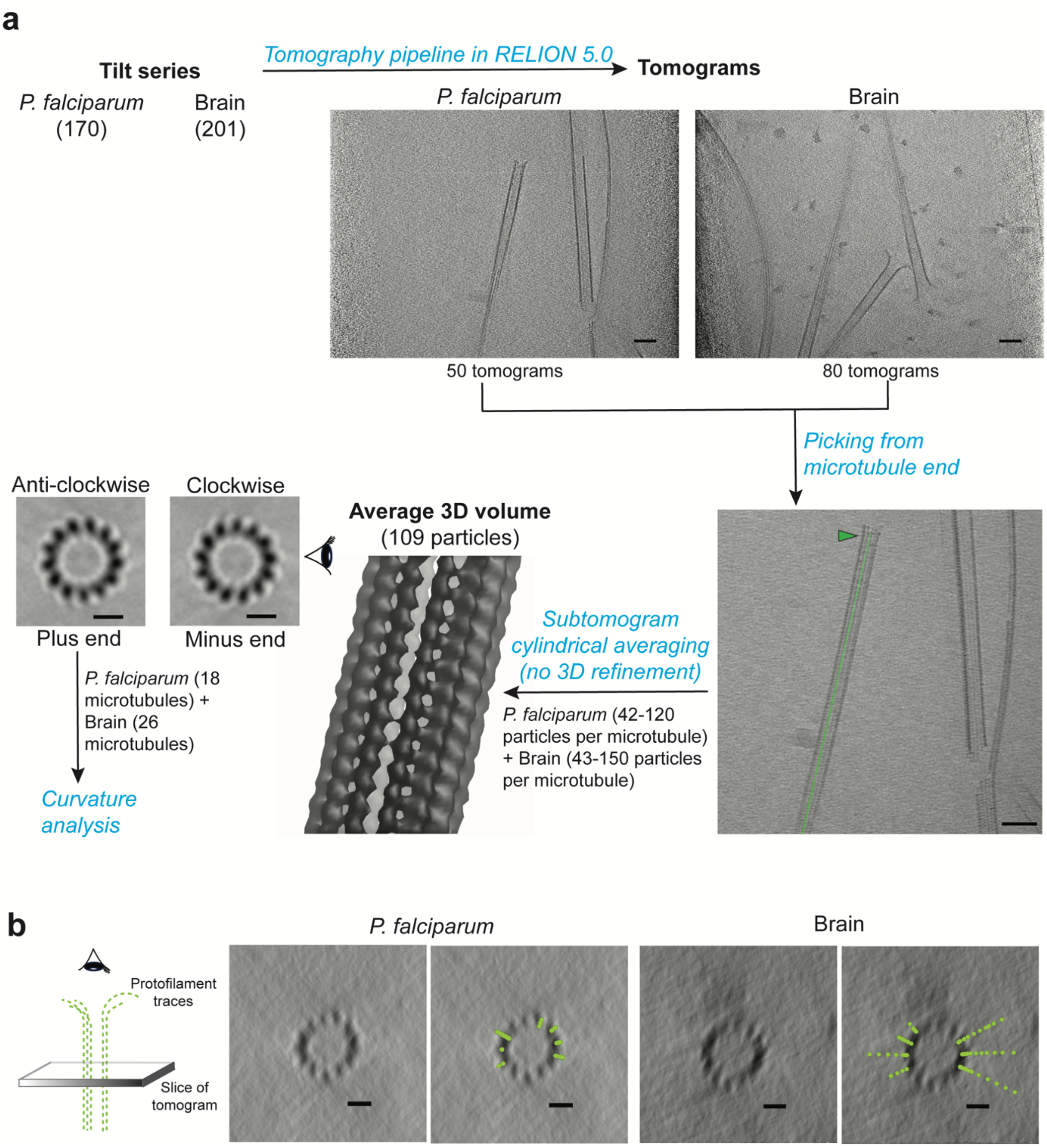

Supplementary Fig. 5: Cryo-ET workflow for processing plus ends of *in vitro* microtubules. a, Slices from representative tomograms have been shown. Scale bar=50 nm. Positions of particles picked along the microtubule at a spacing of 8 nm are shown as green points. Picking was initiated at the microtubule end depicted with a green arrow. Scale bar=50 nm. The average 3D volume obtained from alignment of extracted subtomograms was examined from the microtubule end. The directional tilt of protofilaments was used to assign polarity of the microtubule. Scale bar=10 nm. **b**, Left, schematic of microtubule tomogram transverse view, depicting the tomogram slice and projection of protofilament traces (green dotted lines), indicating that only those protofilaments not affected by the missing wedge were

traced; middle, right, exemplar data for *P. falciparum* (middle) and brain (right, bovine tubulin) paclitaxel-stabilized microtubules, with views ± protofilament traces for comparison. Scale bars=10nm

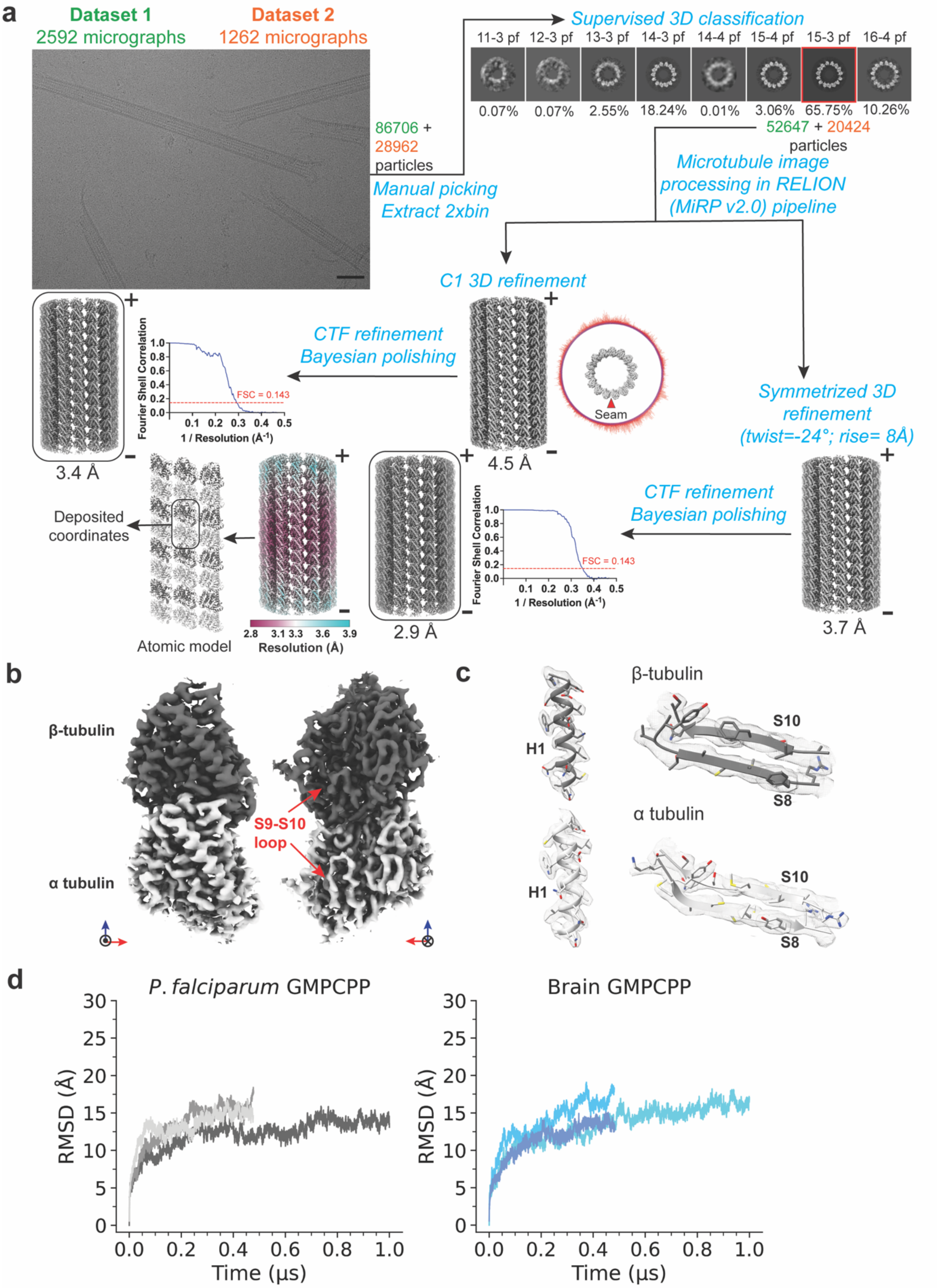

Supplementary Fig. 6: Structural properties of *P. falciparum* 15-3 protofilament GMPCPP microtubules. **a**, Cryo-EM data processing workflow. Steps of the pipeline are indicated in blue. Multiple datasets used during processing are color-coded. A representative

micrograph is shown. Scale bar = 50 nm. Cross-sections of classes obtained from reference-based 3D classification along with their corresponding architectures are shown. EM reconstructions are depicted in grey. The plus and minus ends of the microtubules are labeled. 3D reconstructions that have been deposited to the EMBD are highlighted using round-edge boxes. Cylindrical histogram plots around the microtubule (top view) represent angular distribution of particles used in C1 3D refinement and the seam is indicated with a red arrowhead. Corresponding FSC plots and density coloured by local resolution are also shown. The fitted atomic model is displayed as a cartoon representation, and the deposited coordinates for a tubulin dimer are outlined by a round-edge box. **b,** Cryo-EM density of a *P. falciparum* tubulin dimer segmented from the 15-3 protofilament GMPCPP microtubule reconstruction, viewed from outside (left) and lumen (right). α-tubulin is shown in light grey and β-tubulin in dark grey. **c,** Representative regions of the density depicted as a mesh are shown with the fitted model (cartoon representation with side chains). **d,** RMSD profiles of Cα atoms for three replicate molecular dynamics runs of *P. falciparum* (grey) and brain (blue) GMPCPP microtubules (porcine α1B- and β-tubulin; PDB ID: 6DPU) over 1 μs simulation time. The RMSD stabilises after approximately 300-400 ns for both systems, indicating equilibration of the microtubule structures.

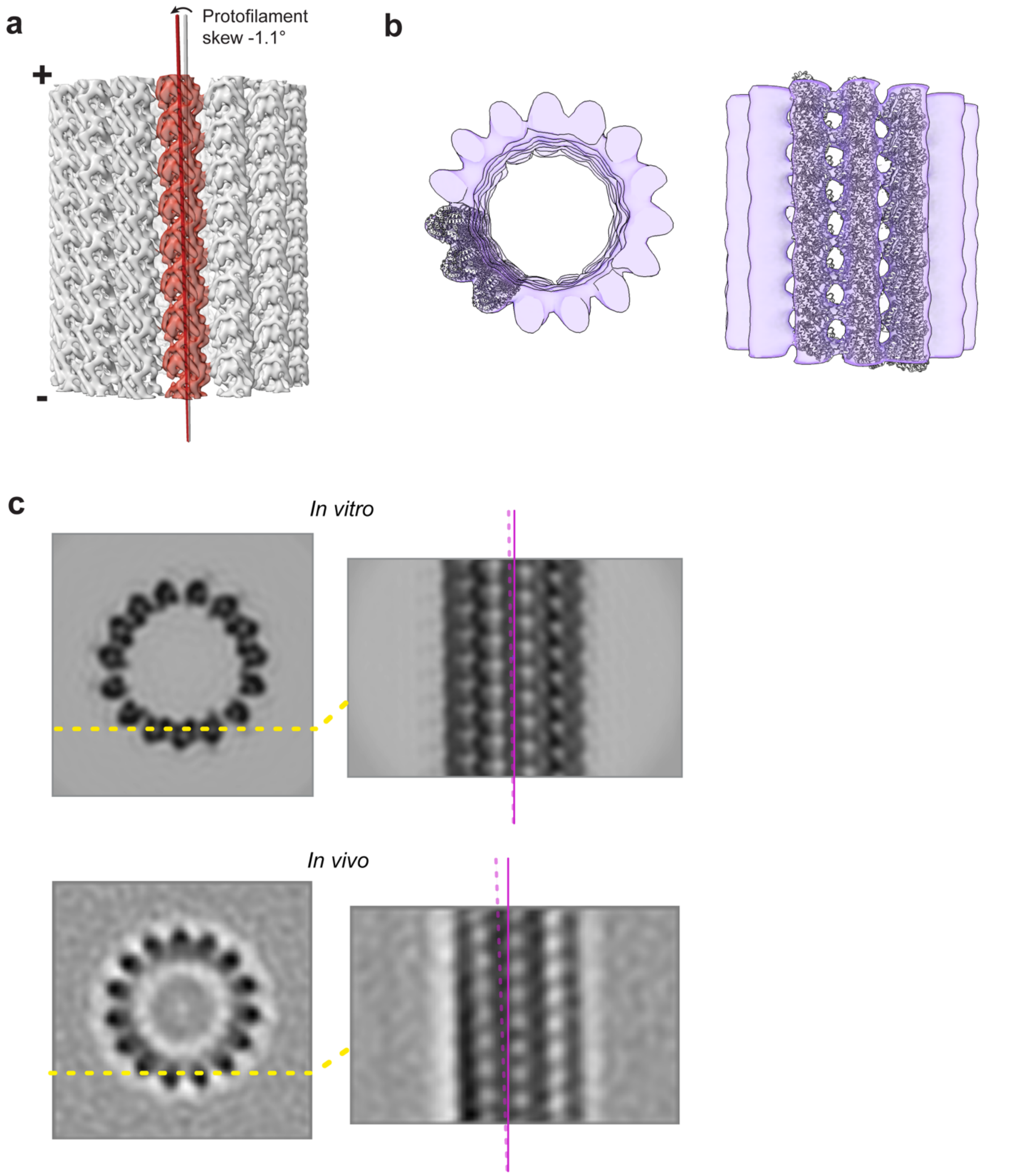

Supplementary Fig. 7: Comparisons between *in vitro* and *in vivo* 15-protofilament *P. falciparum* microtubules. a, Protofilament skew of -1.1° indicated as the angle between the protofilament and microtubule axes. The density for the *P. falciparum* microtubule is shown in grey with a single protofilament coloured in red. Minus and plus ends of the microtubule are shown. **b**, Cross-section and side views of fit of an atomic model of 12 tubulin dimers (3 protofilaments x 4 dimers each) from symmetrized reconstruction of *in vitro* 15-protofilament GMPCPP microtubule in 15-protofilament microtubule obtained from cryo-electron

tomography of *P. falciparum* gametocytes. The model (grey) is depicted in cartoon representation, and the subvolume average is coloured transparent violet. **c,** Depiction of protofilament skew in *in vitro* (top) and *in vivo* (bottom) 15-protofilament microtubules. Cross (left) and longitudinal (right) sections of symmetrized 3D reconstruction and subvolume average are shown for *in vitro* and *in vivo* microtubules respectively. The solid magenta line indicates the microtubule axis while the protofilament axis is marked with a dashed magenta line.
